# Prevalence of severe acute respiratory syndrome coronavirus 2 (SARS‐CoV‐2) and feline enteric coronavirus (FECV) in shelter‐housed cats in the Central Valley of California, USA

**DOI:** 10.1002/vro2.73

**Published:** 2023-10-20

**Authors:** Daniel Chen, Andrés M. López‐Pérez, Karen M. Vernau, David J. Maggs, Soohyun Kim, Janet Foley

**Affiliations:** ^1^ Department of Medicine and Epidemiology School of Veterinary Medicine University of California Davis California USA; ^2^ Red de Biología y Conservación de Vertebrados Instituto de Ecología Xalapa México; ^3^ Department of Surgical and Radiological Sciences School of Veterinary Medicine University of California Davis California USA; ^4^ William R. Pritchard Veterinary Medical Teaching Hospital School of Veterinary Medicine University of California Davis California USA

## Abstract

**Background:**

Non‐human animals are natural hosts for the virus causing COVID‐19 (severe acute respiratory syndrome coronavirus 2 [SARS‐CoV‐2]) and a diversity of species appear susceptible to infection. Cats are of particular concern because of their close affiliation with humans and susceptibility to infection. Cats also harbour feline enteric coronavirus (FECV). Our objectives were to document the prevalence of SARS‐CoV‐2 and FECV in feline populations with high turnover and movement among households in the Central Valley of California, USA.

**Methods:**

A cross‐sectional study of 128 shelter and foster cats and kittens in the Central Valley of California was performed from July to December 2020. PCR was performed on rectal and oropharyngeal samples to detect SARS‐CoV‐2 RNA and on rectal samples to detect FECV RNA.

**Results:**

Among 163 rectal and oropharyngeal fluid samples gathered from sheltered and fostered cats and kittens in central California, SARS‐CoV‐2 nucleic acids were not detected from any cat or kitten. In contrast, FECV nucleic acids were detected in 18% of shelter‐housed cats; 83% of these positive samples were collected from cats housed in adjacent cages.

**Conclusions:**

These data may be helpful when considering the allocation of resources to minimise the harm of FECV and SARS‐CoV‐2 in household pets and shelter environments.

## INTRODUCTION

The COVID‐19 pandemic is considered the most devastating health crisis of the 21st century,^1^ with a mounting toll of more than one million confirmed human deaths worldwide by 2022.^2^ As with many coronaviruses, the natural hosts for the causative agent of COVID‐19 (severe acute respiratory syndrome coronavirus 2 [SARS‐CoV‐2]) are non‐human animals. A diversity of species are susceptible to infection with this virus. Cats are of particular concern because of their close affiliation with humans and because SARS‐CoV‐2 can infect household cats.^3^ In addition to SARS‐CoV‐2, a feline enteric coronavirus (FECV) is endemic in domestic cats, particularly those housed in shelters, which typically produce mild and transient clinical signs.^4^ However, endemic FECV can mutate to cause a clinical syndrome known as feline infectious peritonitis (FIP). This deadly disease of cats is caused by a mutation that allows the coronavirus to enter and replicate in monocytes.^5^ Feline infectious peritonitis is almost always fatal unless treated. Both FECV and SARS‐CoV‐2 virus can be shed in the faeces of cats and humans, respectively,^6^ although SARS‐CoV‐2 is more typically shed from the human respiratory tract.

Throughout the COVID‐19 pandemic, animal shelters continued to serve important roles in society, working to find suitable and permanent homes for unowned animals. However, relinquished, sheltered and fostered cats and kittens may be at high risk for infection with numerous pathogens, including SARS‐CoV‐2 and FECV due to the high turnover rates, proximity to other cats, overcrowding, influx of animals with complicated medical conditions, stress or limited shelter resources. These factors increase cat‐to‐cat, cat‐to‐human and human‐to‐cat contact, possibly exacerbating epidemiological cycles of disease.

The extent to which SARS‐CoV‐2 replicates in vulnerable cat populations, especially in those with high turnover rates and high rates of movement among households or institutions, has yet to be determined. Although cats seem to play a limited role in the spread of SARS‐CoV‐2 and the risk of cats spreading this virus to humans is considered to be low,^7^ infection could potentially lead to low adoption rates and an increased reliance on euthanasia. This study was designed to survey the prevalence of FECV and SARS‐CoV‐2 within cats and kittens in shelters and foster homes in the Central Valley of California.

## MATERIALS AND METHODS

### Study sites

**FIGURE 1:**
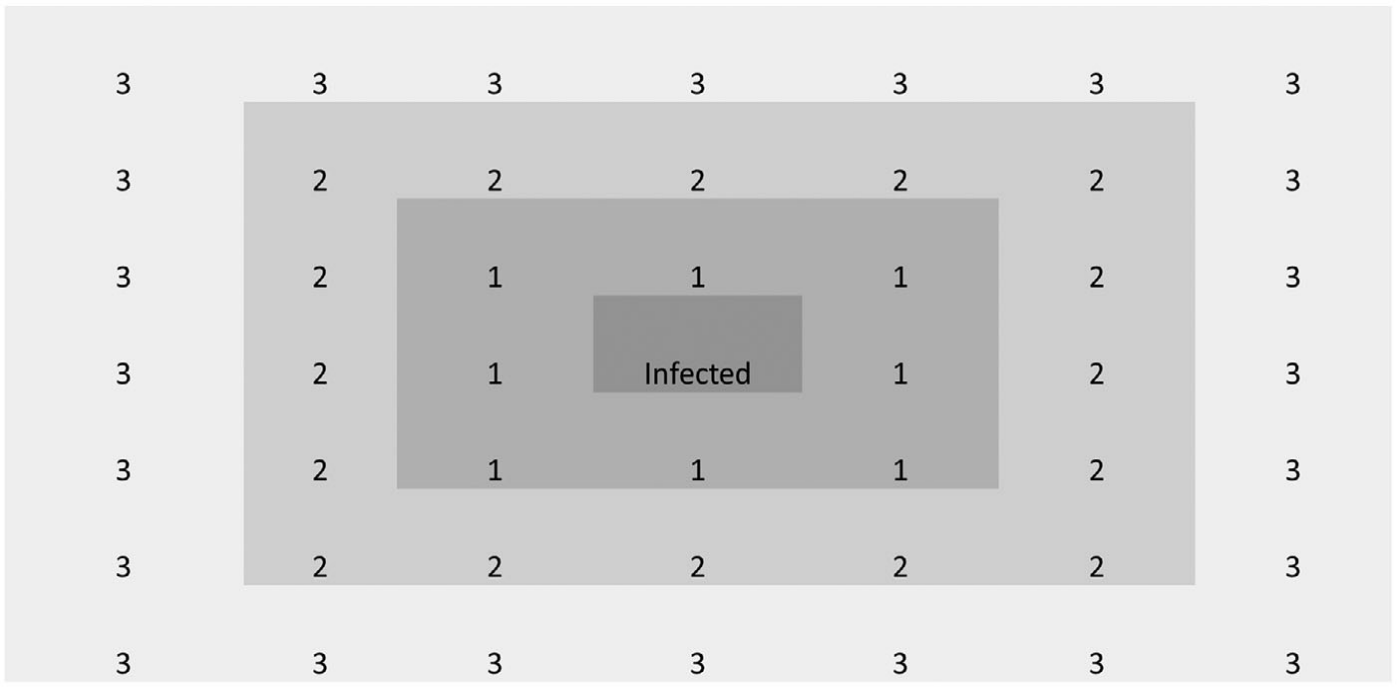
Schematic showing how proximity was scored for cats surrounding a cat with a positive feline enteric coronavirus PCR test result (‘infected’) for cats tested at the City of Stockton Animal Services Center, CA. Numbers refer to the distance score given to each cat: a score of 1 indicated that the cat was housed adjacent to an infected cat, 2 indicated that there was a cage between the cat and an infected cat and 3 indicated that there were two cages between the cat and infected cat.

Samples were collected from cats and kittens at two locations: the City of Stockton Animal Services Center (SAS) and kittens seen at the William R. Pritchard Veterinary Medical Teaching Hospital at the University of California, Davis (UCD). The SAS is an organised animal care facility (‘shelter’) with an annual intake of approximately 11,000 animals. The facility conforms to local animal safety guidelines and rabies quarantine. It provides veterinary care and other pertinent services to the residents of Stockton and San Joaquin County. The cat population was segregated based on their health status and age group. Within each unit, cats were housed separately in stainless steel cages, while kittens were often left in small family groups. Every day, dishes and litter boxes were removed and cleaned in an industrial dishwasher and the cage was wiped down with hot water. On every cat's exit, the cage contents were cleaned in the dishwasher and the cage was sterilised with a 4.25% hydrogen peroxide‐based cleaner. Cats and kittens of all ages were examined by the authors on various days post‐intake (as they were made available for study enrolment). Staff collected rectal and oropharyngeal swab samples for PCR, and they collected data on sex, neutering status, breed, vaccination and health status, cage location within the shelter and age group—as defined by the American Association of Feline Practitioners/American Animal Hospital Association Feline Life Stage Guidelines (kitten [0–6 months], junior [7 months–2 years], adult [3–6 years], mature [7–10 years], senior [11–14 years] and geriatric [15+ years]).^8^ Distance from an infected cat was analysed by assigning to each cat a score from 1 to 4, where a score of 1 indicated that the cat was housed adjacent to an infected cat, 2 indicated that there was a cage between the cat and an infected cat, 3 indicated that there were two cages between the cat and infected cat and 4 indicated that there were three cages between the cat and infected cat (see Figure 1).

At UCD, oropharyngeal samples were collected from kittens diagnosed with ocular disease associated with upper respiratory tract infection at the time of enrollment in another study (protocol 20962). All kittens sampled at UCD were less than 12 weeks of age and less than 3.0 lb bodyweight. They were owned by local 501c3 animal or government‐run animal shelters within 25 miles of UCD. All kittens owned by 501c3 had originally been taken in from government‐run shelters and distributed to the foster families. Because of the diversity of original sources and foster housing in this group, details of management varied and were not available to the authors.

### Sample collection

Sixty‐eight samples from SAS and 93 from UCD were collected at the respective facilities or by foster caregivers at home (two samples). They comprised 35 rectal swabs and 126 oropharyngeal swabs. When samples were obtained by caregivers, a package of sampling equipment was posted to the participant along with instructions. Rectal and faecal samples suitable for FECV testing were not collected from UCD kittens. Samples from UCD kittens were collected upon intake at UCD.

For sample collection, operators wore latex gloves wetted with RNase AWAY surface decontaminant (Thermos Scientific, Waltham, MA, USA). A sterile polyester swab (Puritan, Guilford, ME, USA) was then used to collect samples from the oropharynx. The tip of the swab was then broken off and placed in an RNase‐free Eppendorf tube taking care to avoid contacting the inside or lid of the Eppendorf tube. At SAS, a second sample was collected from the rectum of each cat or kitten. Samples were transported on ice to the laboratory and then subjected to RNA extraction and PCR.

### Molecular diagnosis

RNA was extracted from faeces and oropharyngeal swabs using a commercial kit (Qiagen Blood and Tissue Kit, Valencia, CA, USA). Total RNA was transcribed to cDNA using 5× all‐in‐one RT MasterMix (Applied Biological Materials, Richmond, British Columbia, Canada). RNA extraction and transcription were performed in class II biosafety cabinets and all pipetting was done with plugged tips. All runs included positive (sequence‐confirmed coronavirus‐infected samples) and negative (water) controls.

Real‐time PCR for SARS‐CoV‐2 was performed on rectal and oropharyngeal swabs by amplifying two conserved fragments of two target genes, including open reading frame 1ab (ORF1ab) and nucleocapsid protein (*N)* using published primers,^9^ with the following conditions: 50°C for 2 min followed by 95°C for 10 min and then 50 cycles of 95°C for 15 s and 60°C for 1 min. PCR was performed on a 12‐μL reaction mixture containing 4.4 μL of nuclease‐free water, 6 μL of Master mix (Thermo Scientific), 0.6 μL of primer/probe mix and 100 ng of DNA. Samples were considered positive if the cycle threshold (Ct) value was less than 40 with characteristic amplification plots.

To detect FECV RNA, a conserved fragment of the 7b gene was amplified from rectal samples using primers C202 and 177.^10^ Cycling conditions were as follows: initial denaturation (95°C for 2 min) followed by 35 cycles of 95°C for 30 s, 55°C for 1 min and 72°C for 2 min, followed by a final extension (72°C for 7 min). PCR was performed on a 25‐μL reaction mix containing 12.5 μL Green GoTaq (Promega, Madison, WI, USA), 6.5 μL nuclease‐free water, 2.5 μL 202C primer, 2.5 μL 177 primer and 100 ng DNA. PCR products were visualised on 1% agarose gels stained with Gelstar (Lonza Bioscience, Morrisville, NC, USA) under UV‐transillumination.

### Statistical analysis

The study was structured as a cross‐sectional survey of prevalence of SARS‐CoV‐2. Statistical analysis was performed in R 4.0.3 (R Core Team. R: a language and environment for statistical computing. Vienna, Austria: R Foundation for Statistical Computing; 2020. Available from: www.R‐project.org), with a cutoff of *p‐*value less than 0.05 used to infer statistical significance. Fisher's exact test was used to compare sex ratios between the UCD and SAS populations; logistic regression was used to compare age distribution between sites. For cats at SAS, logistic regression models with binomial distribution were also constructed to evaluate the association of the presence/absence of FECV infection (response variable) with sex, age, breed, neuter status and whether or not the cat was immunised against herpesvirus, calicivirus and panleukopenia virus as explanatory variables. A Wilcoxon rank sum test with continuity correction was performed to analyse whether cats that were, or were not, FECV PCR‐positive were likely to be in proximity to other FECV PCR‐positive cats.

## RESULTS

### Descriptive statistics

A total of 128 cats were sampled, including 33 from SAS and 95 from UCD. Overall, at both sites, 48.4% of the sampled cats were female. The sex ratios at SAS (1 female:1 male) were not significantly different (*p* = 0.42) from those for kittens at UCD (0.96:1). Considering age, 82.8% were kittens, 13.3% were juniors, 1.6% were adults and 2.3% were mature. While the UCD group sampled kittens only, the SAS group included cats of multiple age classes, with significantly (*p* = 0.87 × 10^−6^) more junior cats at SAS than at UCD. In total, 42.2% of cats were neutered (all at SAS because kittens from UCD were too young to undergo neuter surgery). There was documentation of vaccination for 40.6% of the cats or kittens (Table 1). All kittens of sufficient age were inoculated with a feline viral rhinotracheitis, calicivirus and panleukopenia vaccine upon intake. Rabies vaccination was not practised at SAS and all UCD kittens were too young for vaccine.

**TABLE 1:** Breed, sex, age distribution, percent vaccinated against feline viral rhinotracheitis (FVR), feline calicivirus (FCV) and feline panleukopenia (FPV) and percent neutered, for two populations of cats in the Central Valley of California (USA) in 2020.

	Stockton Animal Services			University of California, Davis		
Number sampled	33			95		
Percent female	48.5			48.4		
Percent male	51.5			51.6		
Breeds	One each of Bengal, Russian blue and Siamese; 30 DSH			2 DLH, 5 DMH, 83 DSH & 5 Siamese		

Neuter and vaccine status	Number	% FVR—vaccinated	% neutered	Number	% FCV and FPV—vaccinated^a^	% neutered
Kitten (0‐6 months)	12	100	0	93	36.6	0
Junior (7 months‐2 years)	11	100	50	2	100	100
Adult (3‐6 years)	7	100	100	0	N/A	N/A
Mature (7‐10 years)	3	100	100	0	N/A	N/A

*Note*: N/A indicates not applicable for that category.

Abbreviations: DLH, domestic long‐hair cat; DMH, domestic medium‐hair cat; DSH, domestic short‐hair cat.

^a^ Most kittens in University of California, Davis, foster network were too young for some vaccines and neutering. Ages are classed as described in section ‘Materials and Methods’.

SARS‐CoV‐2 viral DNA was not detected by PCR in any of the 163 samples. In contrast, FECV RNA was detected in samples from six of 34 cats (17.65%) housed at SAS. Using univariate logistic regression models, none of the predictors (age, sex, breed, immunisation history or neuter status) was significantly associated with the detection of FECV RNA (Table 2).

**TABLE 2:** Results from logistic regression with binomial distribution for predictor variables and their association with feline enteric coronavirus PCR results in cats and kittens at the City of Stockton Animal Services Center in the Central Valley of California, USA, in 2020.

Predictor	Estimated standard deviation	Error	*z*‐Value	Pr > *z*
Sex	0.8362	0.9453	0.885	0.3764
Neuter status	0.5108	1.2543	0.407	0.6838
Age group (junior)	0.9808	1.3017	0.753	0.451
Age group (adult)	1.3863	1.3229	1.048	0.295
Age group (mature)	2.4849	1.7559	1.415	0.157
Vaccination	0.8362	0.9453	0.885	0.3764
Breed	0.9555	1.3185	0.725	0.4686

**FIGURE 2:**
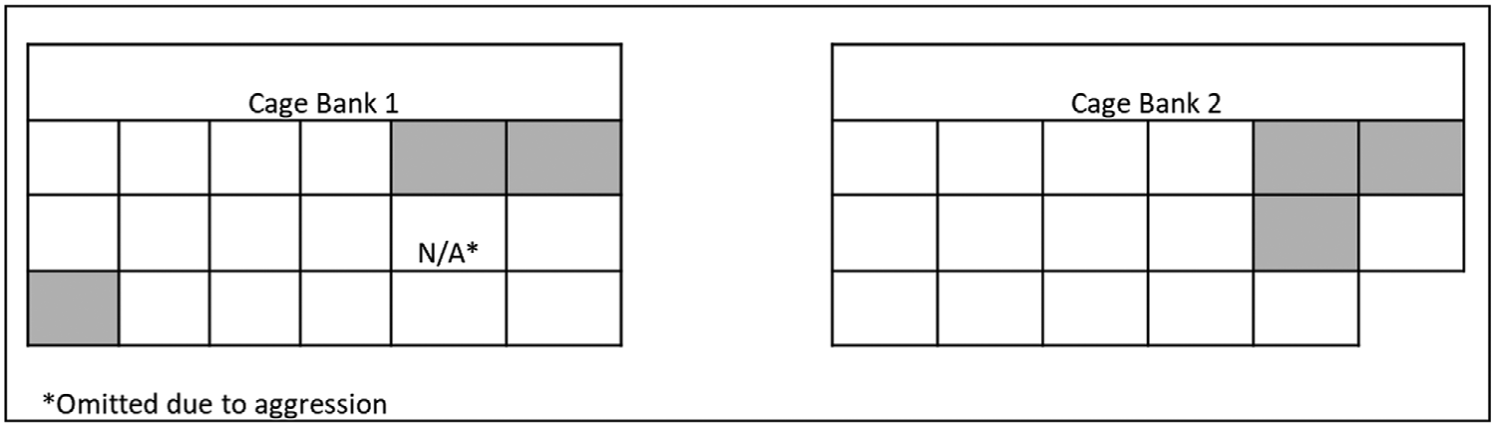
Chart of locations in Stockton Animal Shelter of cats that were feline enteric coronavirus PCR positive (grey) or negative (white).

Cats in the Stockton shelter were housed in one of two banks of cages. As shown in Figure 2, five of six cats from which FECV RNA was detected were in adjoining cages. Although not statistically significant (*W* = 53, *p* = 0.16), the median proximity index (MPI), as described in Figure 1, tended to be lower among PCR‐positive (1.5 MPI) than PCR‐negative cats (1.9 MPI).

## DISCUSSION

This study aimed to provide insight into the risk of SARS‐CoV‐2 infection among cats and kittens in high‐turnover environments in the Central Valley of California during 2020 using rectal and oropharyngeal samples, as well as to perform an updated prevalence survey for an endemic FECV using rectal samples from cats in a Californian animal shelter. Although SARS‐CoV‐2 RNA was not detected in any cats or kittens sampled, there was a considerable presence of FECV in cats within the shelter.

The fact that SARS‐CoV‐2 RNA was not found in samples from foster or shelter cats may alleviate some reservations potential adopters may have regarding taking cats into their household and, thereby, lessen the burdens of shelters. However, there have been several cases of SARS‐CoV‐2 infection in pet animals, including an infected dog and two cats in New York, a cat in Belgium, a cat in Seoul and two dogs in Hong Kong.^11^, ^12^ In all pet cases, the infection was detected only by testing of the upper respiratory tract, although faecal shedding of SARS‐CoV‐2 has also been observed in humans.^13^ In cats experimentally challenged with SARS‐CoV‐2, viral RNA was detected in respiratory and small intestinal tissues when cats were subsequently sacrificed.^14^ Although cat‐to‐cat transmission has been documented,^15^ all of the pet cases appear to have originated from transmission from infected owners or handlers.^16^ One study reported strong evidence for transmission of SARS‐CoV‐2 from cats to humans.^17^ While cats are susceptible to infection with SARS‐CoV‐2, more research is needed to better define the extent of infection in cat populations, attributable clinical signs and the potential for transmission between cats and other animals within the context of the COVID‐19 pandemic and for probable circulation in the future, if any.

A prevalence survey of FECV in a shelter was performed, in part as a proof of concept that we could detect other circulating coronaviruses (SARS‐CoV‐2) in the target populations. Due to early permitting concerns, faecal samples suitable for FECV testing could not be collected from foster cats. Wild‐type FECV causes few or no clinical signs but often proves to be fatal when the virus mutates to the FIP virus,^18^ although new treatments are showing promise.^19^ High FECV loads and thus likelihood of FIP‐associated mutations are more common when cats are in multi‐cat environments.^4^, ^20^ In addition, certain breeds may be at greater risk of developing FIP.^21^ A survey from Davis, California, reported a seroprevalence of 20% in pet cats living in private households and 87% in purebred cats living in catteries.^22^ Our study used PCR, thus allowing us to detect active infection (rather than immunological evidence of prior exposure as with serology) and showed that active FECV infection was common in the shelter. Five of the six infected cats were in neighbouring cages, highlighting the need for robust infection management strategies.

The two populations in our study had important distinctions. Although most kittens in the UCD population originated in animal shelters, they were younger than the SAS population and were distributed in a network of homes; they all initially presented with respiratory tract and ocular infections because they were concurrently enrolled in another study. In contrast, at SAS, cats of all ages were housed in individual cages within a single large facility.

There were some important limitations to our study. These included the differences in the populations, the lack of background data available on the cats and the variability in management of the cats prior to the time of sampling. Moreover, the two populations tested may not be representative of all shelter cat populations, where there may, for example, be differences in exposure and housing density. Cats in the surveyed shelter were sampled as available. They were of various ages and had been in the shelter for differing durations and information about whether they were healthy or had some concurrent illness, was not available to study staff. Because of the nature of the kitten rescue organisations, kittens may have been co‐housed with littermates or other similarly aged kittens, with or without the queen, and possibly from a shelter and through one or several foster homes. Furthermore, the fact that cats in the foster network were all kittens and presented with ocular and respiratory diseases could also impact our results and the probability of being exposed to SARS‐CoV‐2 or other viral pathogens. However, a previous report of SARS‐CoV‐2 showed no difference in prevalence between cats with and without clinical evidence of respiratory disease,^7^ suggesting that a difference would not have been expected between the groups assessed in the present study.

Another shortcoming was that rectal swabs and faecal samples suitable for FECV testing were not collected from UCD kittens. Due to the small sample size for FECV and sampling limitations, caution should be exercised when extrapolating this information to the overall population of adoptable cats. With a larger sample size, knowing that so few cats and kittens in the community and available for adoption were infected with SARS‐CoV‐2 should reduce concern about cat adoption contributing to this serious public health risk. Despite the study limitations, this study provides no evidence that SARS‐CoV‐2 was present in cats and kittens housed in shelter or foster homes. Knowing the prevalence of FECV is important for development of shelter protocols such as quarantine, housing and cleaning, as well as guiding adopter and staff expectations of cats developing FIP.

## AUTHOR CONTRIBUTIONS

All authors contributed to the study design. Sample collection was done by Karen Vernau, David Maggs, Andrés M. López‐Pérez and Daniel Chen. Material preparation and analysis were performed by Daniel Chen and Andrés M. López‐Pérez. The first draft of the manuscript was written by Daniel Chen and all authors commented on previous versions of the manuscript. All authors read and approved the final manuscript. Janet Foley managed the laboratory, permitting and funding.

## CONFLICTS OF INTEREST STATEMENT

The authors declare they have no conflicts of interest.

## ETHICS STATEMENT

This project was conducted with informed consent and approval by the UC Davis Institutional Animal Care and Use Committee under protocol number 21301.

## Data Availability

Data sharing not applicable—no new data generated, or the article describes entirely theoretical research.
